# Late diagnosis of Marfan syndrome with fatal outcome in a young male patient: a case report

**DOI:** 10.4076/1757-1626-2-8827

**Published:** 2009-09-09

**Authors:** Aurora Bakalli, Tefik Bekteshi, Merita Basha, Afrim Gashi, Afërdita Bakalli, Petrit Ademaj

**Affiliations:** 1 Department of Cardiology, Internal Clinic, University Clinical Center of Kosova, Rrethi i spitalit, p.n. 10000 Prishtina, Kosova; 2 Department of Radiology, University Clinical Center of Kosova, Rrethi i spitalit, p.n. 10000 Prishtina, Kosova; 3 Department of Rheumatology, Internal Clinic, University Clinical Center of Kosova, Rrethi i spitalit p.n. 10000 Prishtina, Kosova; 4 Department of Ophthalmology, University Clinical Center of Kosova, Rrethi i spitalit p.n. 10000 Prishtina, Kosova; 5 Department of Cardiology, Internal Clinic, Regional Hospital of Peja, Rrethi i spitalit p.n. 30000 Peja, Kosova

## Abstract

**Introduction:**

Marfan syndrome is a heritable disorder of the connective tissue that affects many organ systems. However, the most serious complication in patients with Marfan syndrome is progressive aortic root dilation, which may lead to aortic dissection, rupture or aortic regurgitation. Prevention of these life threatening complications is of major importance.

**Case presentation:**

We report here a case of a 34-year-old, Caucasian male diagnosed for the first time with Marfan syndrome. He required medical attention due to his chest pain that resulted as a consequence of strenuous physical effort. Medical examinations revealed severe aortic root enlargement and aortic intramural hematoma. Patient ended-up fatally during open heart surgery.

**Conclusion:**

It is very important to recognize on time Marfan syndrome, as preventive actions that should be undertaken can avoid its serious consequences.

## Introduction

Marfan syndrome is a heritable disorder of the connective tissue, with primary involvement of musculoskeletal, cardiovascular, and ocular systems. The most serious complication in patients with Marfan syndrome presents progressive aortic root dilation that may lead to aortic dissection, rupture or aortic regurgitation, which used to be the main cause of death in this patient category prior to the era of successful preventive therapies. Preventive therapy includes regular imaging of the aorta in order to assess progression of aortic enlargement, prescription of Beta blocker therapy and prophylactic aortic repair when aorta reaches a sufficient size to threaten dissection, rupture or serious aortic regurgitation [1].

We report here a case of a 34-year-old male diagnosed for the first time with Marfan syndrome, with severe aortic root enlargement and aortic intramural hematoma, which ended up fatally during open heart surgery.

## Case presentation

A 34-year-old Albanian, Kosovan male was sent to our centre to undergo echocardiography, in order to clarify the unexplained chest pain that had started two days earlier. Patient explained that his chest pain started after physical exertion, which consisted of carrying wood. This pain radiated to the back and was of strong intensity. He was visited by an internal medicine doctor and was then referred to the cardiologist.

Patient reported that this was the first time to experience such pain. He had been suffering from bronchial asthma since childhood. Patient was not aware of suffering from other diseases, nor was he aware of serious diseases or sudden deaths among his family members. His parents did not show features of the Marfan syndrome and the patient did not have other siblings.

**Figure 1:**
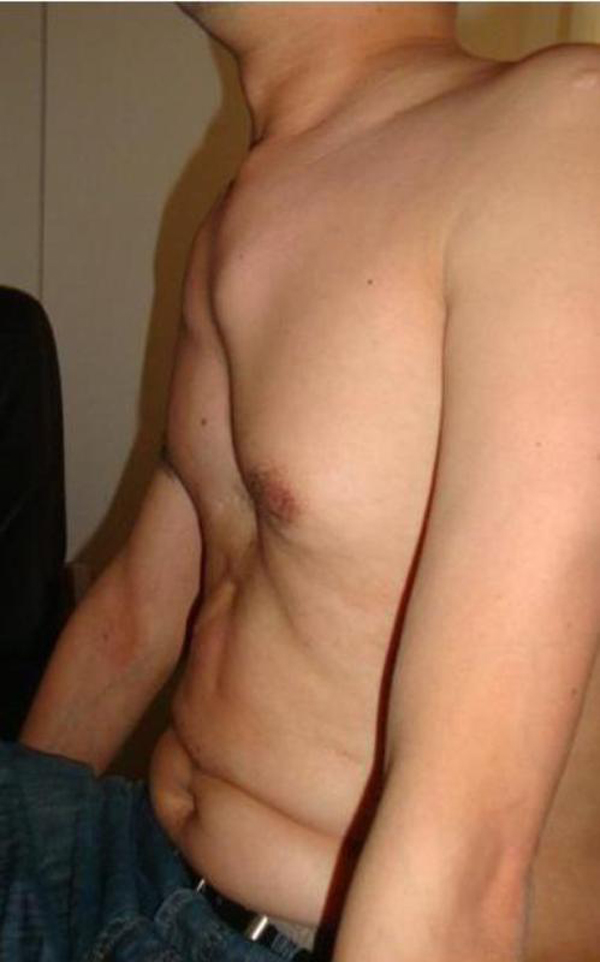
**Pectus excavatum**. Image shows anterior chest wall deformity, pectus excavatum.

He was 176 cm tall and weighed 61 kg, had elongated arms, arachnodactyly and moderate joint laxity. Patient had serious chest wall deformities, which included thoracic scoliosis of the vertebral column and pectus excavatum (Figure 1). On auscultation of the heart, a diastolic murmur 1-2/6 over the aortic area was heard. Auscultation of the lungs demonstrated prolonged expiration and mild wheezing. Blood pressure was 150/90 mmHg. ECG displayed sinus rhythm, heart rate of 80/minute, without any abnormalities.

Chest X-ray revealed osseous deformities previously mentioned and a heart size that encompassed almost entire left hemi-thorax.

**Figure 2:**
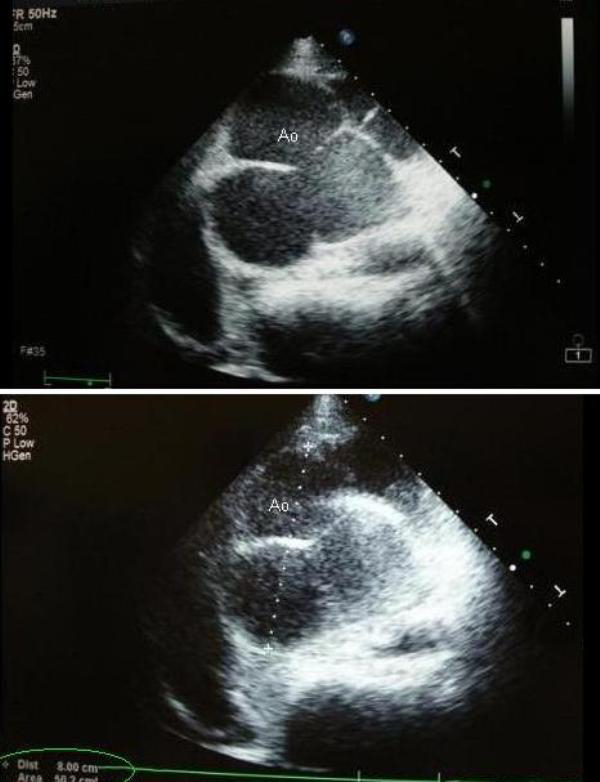
**Echocardiography image of aorta**. **(A)** Transthoracic echocardiography image of aorta, on parasternal short axis view, shows a dilated aorta at the level of aortic valves, measuring 80 mm **(B)**.

**Figure 3:**
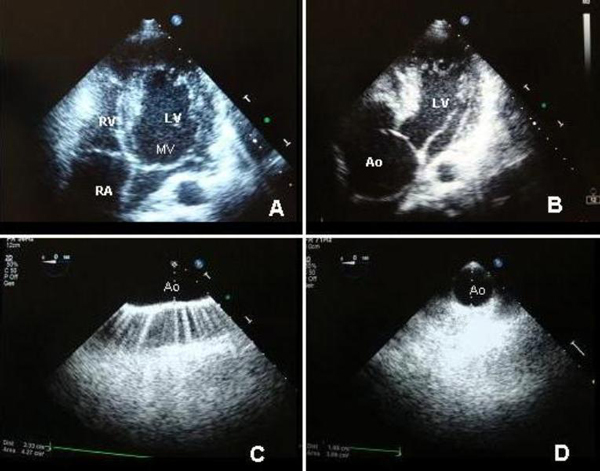
**Tansthoracic and transesophageal echocardiography images**. **(A)** Four chamber apical view demonstrating mitral valve prolapse of the anterior leaflet. **(B)** Apical view of the heart showing the large size of the aorta compared to the left ventricle. **(C)** Transesophageal echocardiography presents a normal size aortic arch and **(D)** descending aorta.

Transthoracic echocardiography showed enormous size of the aortic bulb, measuring 80 mm on short axis parasternal view at the level of aortic cusps (Figure 2). Despite the very large size of the aortic root, aortic regurgitation was mild. Mitral valve prolaps of the anterior leaflet with mild mitral regurgitation was also noted (Figure 3A).

Transesophageal echocardiography confirmed the massive size of the aortic root and normal size of the aortic arch and descending thoracic aorta (Figures 3C and 3D). We were unable to detect signs of aortic dissection, thus computed tomography (CT) of the chest was recommended.

**Figure 4:**
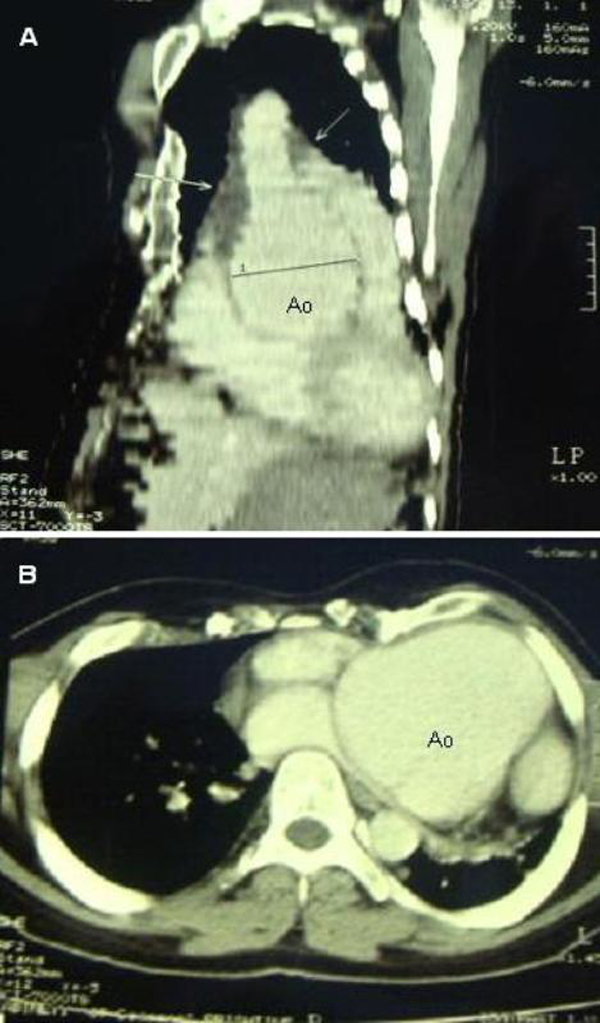
**Chest CT**. CT image in sagittal orientation showing a very large aortic root and intramural hematoma, which is depicted by arrows **(A)**. Transverse CT image demonstrates a large aortic bulb and heart structures encompassing almost entire left hemi-thorax **(B)**.

Contrast CT of the chest demonstrated severe enlargement of aortic root measuring 78.7 mm, and intramural hematoma that reached the aortic arch (Figure 4).

An ophthalmologic examination discovered the presence of myopia, of -3 diopters. No signs of ectopia lentis or retinal ablation were found. Ocular pressure was 12 mmHg.

In accordance with Ghent criteria, patient was diagnosed as having Marfan syndrome, complicated by severe aortic root aneurysm and type A intramural hematoma with no intraluminal tear or flap being detected. As a result, patient was put on Beta blocker therapy and was referred immediately for surgical intervention, replacement of the affected aorta with composite aortic valve- ascending aortic conduit, in a specialized cardio-surgery centre abroad. Unfortunately, patient ended up fatally during the open heart surgery.

## Discussion

The most life threatening complication of Marfan syndrome is aortic aneurysm which can lead to aortic dissection, rupture or both. Accordingly, echocardiography examination with special emphasis on evaluation of ascending aorta and heart valves is mandatory in patients with Marfan syndrome. The severity of aortic disease is in relation to the extent of aortic dilation, the length of the dilated segment and the location of the aortic involvement [2]. Echocardiogram is recommended six months following the initial examination to determine the rate of aortic dilation and afterwards annually if aortic size is stable. In instances of aortic dilation of more than 45 mm, more frequent imaging of the aorta should be considered [3]. Most of the patients with Marfan syndrome present with enlargement of the ascending aorta or type A aortic dissection, therefore serial examination with transthoracic echocardiography is focused mainly on assessing this portion of aorta.

Management and treatment of the aorta in Marfan syndrome patients should be based on regular imaging of the aorta to evaluate its size and to determine the eventual dilation and its progression; administration of Beta adrenergic receptor antagonist therapy; and prophylactic aortic repair when aorta reaches a size that may threaten dissection or aortic regurgitation. In the pre open-heart surgery era, the average life expectancy of patients with Marfan syndrome was 45 years [4], whereas in our time, owing to preventive measures, it has extended up to 70 years [5]. Beta blockers are recommended in patients with Marfan syndrome mainly to delay aortic root enlargement.

However, studies suggest that beta-blockers must be given early in the course of the disease, when aortic diameter has not exceeded 40 mm, in order to achieve beneficial results [6]. Prophylactic aortic root replacement in patients with Marfan syndrome is recommended when aortic diameter reaches 50 mm, whereas cases that surgery is indicated below this size include identification of rapid aortic growth, presence of more than mild aortic regurgitation, and family history of premature aortic dissection [7]. Exercise restriction represents another preventive approach in patients with Marfan syndrome. These patients are recommended to avoid exhausting exercise activity, particularly isometric exercises [8]. Our patient, regrettably, was not diagnosed on time with Marfan syndrome, thus the above mentioned preventive measures were not applied on him. He experienced his first symptoms of chest pain precisely following isometric physical activity.

Aortic intramural hematoma, a variant of aortic dissection, was detected by CT in our patient. Intramural hematoma of aorta, itself, carries a high lethal risk, with higher mortality rates at the level of aortic root and ascending aorta. According to data obtained from International Registry of Aortic Dissection (IRAD), mortality rate for intramural hematoma relating to ascending aorta was 42.9% with surgical therapy and 33.3% with medical therapy [9].

Early recognition of clinical findings in different organ systems in patients with Marfan syndrome is of major value in order to prevent serious consequences of this disorder. Ghent criteria represent the standard for diagnosing Marfan syndrome in accordance with clinical sings and family history [10]. Diagnosis is made if major criteria are identified in at least two different organ systems and involvement of a third organ system is noted. If family history for Marfan syndrome is positive or FBN1 mutation has been found, then a major criterion in one organ system and involvement of another organ system is necessary for diagnosis.

## Conclusion

It is very important to recognize early Marfan syndrome. When recognized on time, preventive actions should be undertaken in order to avoid life-threatening consequences of this disorder.

## Abbreviations

CT: computed tomography; ECG: electrocardiography; IRAD: International Registry of Aortic Dissection.

## Consent

Written informed consent was obtained from the patient's parent for publication of this case report and accompanying images. A copy of the written consent is available for review by the Editor-in-Chief of this journal.

## Competing interests

The authors declare that they have no competing interests.

## Authors' contributions

AB and TB examined, analyzed and interpreted patient data regarding the aortic pathology. AB was a major contributor in writing the manuscript, also. MB analyzed and interpreted the CT findings. AG examined the musculoskeletal abnormalities and collected relevant papers on musculoskeletal changes on Marfan patients. AB performed the ophthalmologic examination and offered relevant papers on ocular abnormalities on Marfan patients. PA was the first physician that visited and examined the patient at the onset of chest pain and referred him to us with high suspicion of aortic dissection. PA contributed on collecting the relevant papers regarding this case, as well. All authors read and approved the final manuscript.
